# Green Space Attachment and Health: A Comparative Study in Two Urban Neighborhoods

**DOI:** 10.3390/ijerph121114342

**Published:** 2015-11-12

**Authors:** Yang Zhang, Terry van Dijk, Jianjun Tang, Agnes E. van den Berg

**Affiliations:** 1 Department of Spatial Planning & Environment, Faculty of Spatial Sciences, University of Groningen, Landleven 1, 9747 AD Groningen, The Netherlands; E-Mail: t.van.dijk@rug.nl; 2 Centre for Public Health, Queen’s University Belfast, Belfast BT12 6BJ, UK; E-Mail: j.tang@qub.ac.uk; 3 Department of Culture Geography, Faculty of Spatial Sciences, University of Groningen, Landleven 1, 9747 AD Groningen, The Netherlands; E-Mail: a.e.van.den.berg@rug.nl

**Keywords:** urban green spaces, wellbeing, availability, place attachment

## Abstract

The positive relationships between urban green space and health have been well documented. Little is known, however, about the role of residents’ emotional attachment to local green spaces in these relationships, and how attachment to green spaces and health may be promoted by the availability of accessible and usable green spaces. The present research aimed to examine the links between self-reported health, attachment to green space, and the availability of accessible and usable green spaces. Data were collected via paper-mailed surveys in two neighborhoods (*n* = 223) of a medium-sized Dutch city in the Netherlands. These neighborhoods differ in the perceived and objectively measured accessibility and usability of green spaces, but are matched in the physically available amount of urban green space, as well as in demographic and socio-economic status, and housing conditions. Four dimensions of green space attachment were identified through confirmatory factor analysis: place dependence, affective attachment, place identity and social bonding. The results show greater attachment to local green space and better self-reported mental health in the neighborhood with higher availability of accessible and usable green spaces. The two neighborhoods did not differ, however, in physical and general health. Structural Equation Modelling confirmed the neighborhood differences in green space attachment and mental health, and also revealed a positive path from green space attachment to mental health. These findings convey the message that we should make green places, instead of green spaces.

## 1. Introduction

Since the ground breaking work of Kaplan and Kaplan [1], a burgeoning body of literature has emerged on the health benefits of contact with nature. Much of this literature has focused on urban green spaces, such as parks, urban forests and community gardens, as a readily available type of nearby nature with a high potential for health and well-being [2,3]. Epidemiological studies have demonstrated that the presence and the amount of urban green space are related to public health and health-related outcomes like lower mortality and morbidity rates [4,5], decreased levels of stress [6] and increased social interactions [7,8]. The presence of green spaces has also been deemed to stimulate physical exercise [9], but relationships between urban green space and physical exercise have been inconsistent and contradictory [10].

The emergence of a new “green health paradigm” in people-environment studies is to a large extent due to the increased availability of Geographical Information Systems (GIS), which enables the objective measurement of green space indicators for large areas. Although some studies have used proximity indicators like distance to the nearest green space from the home [11], the most widely used GIS-indicator is the percentage of green space in the land cover of a certain territory around people’s homes. Sometimes the territory is defined as a radius around a respondent’s home (see, for example [12,13]) and sometimes as a predefined administrative territory such as a census areas (see, for example [14,15]). Although this geo-statistical approach regards green spaces as flat surfaces in space, the advantages of the method are obvious. It takes advantage of the spatial patterning in the data, and it does not carry the risk of same source bias [16].

Importantly, the evidence for associations between the amount of urban green space and health has contributed to establishing quantity standards in green space provision in various countries. For instance, 2.78 hectares of green spaces per 1000 inhabitants is recommended in Bristol’s Parks and Green Space Strategy [17], and European Environment Agency (EEA) also suggests that the forthcoming EU policy should include “a hierarchy of green spaces to be available within a certain catchment per head of population…” [18]. By the year of 2020, Beijing aims to provide 44%–48% of green spaces in urban area and 40–45 m^2^ green spaces for per person [19]. 

However, an important limitation of the current evidence base for relations between urban green space and health is that green space effectiveness may not be in its quantities but in the qualities of green space (e.g., accessibility, maintenance *etc.*) that can facilitate users’ behaviors and stimulate the interactions between users and the green space. In general, the health benefits of urban green spaces can be expected to increase with increasing accessibility and usability of the nearby green spaces (e.g., [20]). More specifically, it can be argued that the availability of high-quality green spaces that are accessible and usable will enable residents to form an affective bond with their local green spaces and develop a sense of place attachment that is conducive to health and wellbeing [21].

The present research was designed to empirically test the idea that this availability of accessible and usable urban green spaces has additional predictive value for the health effects of green space, along with the mere presence or amount of green space. In the next paragraphs, we will first review previous research on the importance of green space availability for health and wellbeing, followed by a discussion of place attachment as a potential pathway leading from green space availability to health. Next, we will present the method and results of a survey among inhabitants of two Dutch neighborhoods that were similar in the amount of urban green space, but differed in the availability of accessible and usable of green space.

### 1.1. Green Space Availability and Health

Several researchers have pointed out that besides the quantity of green space, quality indicators should also be taken into account when studying the health benefits of green space [20,22]. Broadly, two types of quality indicators can be distinguished: aesthetics indicators, such as naturalness, variety, and landscape type, and use indicators, such as accessibility, usability, number of recreation facilities, and safety [22]. Use indicators may be of especially crucial importance. Indeed, when a space is mapped as “green” but inaccessible, not walkable, invisible or otherwise unusable, it may not even exist in the mental maps of residents. In the present study, we will use the word “availability” to refer to the circumstance that a green space is present and can be actually experienced by people. This implies that a green space is accessible and usable for whoever chooses to visit the place. By taking availability into account, urban green spaces not only exist on maps, but may also play a meaningful role in people’s lives.

There is some evidence for the role of green space availability in the promotion of human health [23]. For example, a study in Florida found that the accessibility of green spaces, but not the mere amount of green space, was associated with decreases in all-cause mortality and mortality from cardiovascular diseases [14]. A Danish study found that usability, as indicated by the presence of walking and cycling routes, was positively related to physical activity in the nearest green space, but not to physical activity levels in general [24]. These latter findings suggest that relationships between green space availability and public health involve more than an increase in general physical activity levels. Indeed, as noted by Ward Thompson and Aspinall [25] “natural open space offers opportunities for peace, relaxation, and social activities and, for many, physical activity is a secondary benefit, rather than a primary purpose in visits”.

In urban neighborhoods, green space availability may also contribute to place attachment as a vital indicator of residents’ health and well-being. Thus far, however, green health researchers have paid little attention to place attachment as a possible pathway leading from green space availability to health. In the following paragraphs, we will discuss the concept of place attachment and its relationships with green space and health.

### 1.2. Place Attachment

The concept of place attachment has drawn attention from environmental psychology [26], human geography [27,28], urban planning [29], and natural resource management [30,31]. In this paper, the working definition of place attachment is derived from environmental psychology and human geography. It describes the emotional attachment between people and places where people endow values by steady accretion of sentiment [27].

Human geographers have since long emphasized that attachment to places helps to foster a sense of wellbeing [32] (p. 2). It is assumed that a person requires a sense of belonging, self-esteem and security through feelings of attachment to places [33] and often unconsciously longs for the places where he or she feels attached [33,34]. Place attachment has been empirically linked to health and wellbeing (see, for example, [35,36,37]). For example, a survey among 443 first-year undergraduates of an Italian university indicated that attachment to neighborhoods and cities positively impacts social wellbeing in terms of five dimensions including social integration, acceptance, contribution, actualization and coherence [36]. A case study of four communities in Pennsylvania revealed that greater community attachment results in better perceived wellbeing [37]. Furthermore, the strength of place attachment has been linked to physical predictors [38]. Certain physical features of a place can make it easier for people to become attached to that place [39]. For instance, in a case study in a retirement community, physical features such as close walking distance to the central activity building and better access to outdoor garden space were found to be essential physical features of place attachment, because they may support social interactions [40]. In terms of green spaces, their availability may be of key importance for people to foster a sense of attachment. However, limited research has tied place attachment to the availability of green space, and measured the impact of green space attachment on health and wellbeing.

### 1.3. Green Space Attachment: A Four-Dimensional Concept

Research has shown that the majority of people’s favorite places consist of natural places [41]. These findings may be interpreted as a manifestation of biophilia, or an innate predisposition to affiliate with natural places and other life-like processes [42,43]. Given people’s strong tendency to connect with nature, it seems appropriate to distinguish “green space attachment” as a special form of place attachment that is highly significant to people. Indeed, previous research suggests that attachment to natural places is a more powerful predictor of positive outcomes like pro-environmental behaviors than attachment to the social, civic elements of a place [44]. In order to capture the full scope of residents’ attachment to urban green spaces in the neighborhood environment, we adopt a multi-structural concept of place attachment, which includes place dependence, affective attachment, place identity and social bonding [31,45,46].

Place dependence can be described as functional attachment to a place, related to the quality of the place in satisfying occupants’ needs and ambitions and how well it serves over alternative places [47]. This functional attachment may be strongly associated with the perception of the unique characteristics of a place for hiking, camping, fishing, scenic enjoyment *etc.* [48]. In terms of green spaces, place dependence represents how residents depend on urban green space in their neighborhood environment for activities and behavioral tendencies, and are less willing to use other green spaces.

Affective attachment has been conceptualized to describe the affective bonds between people and their environment through interaction [49]. Tuan [50] developed the concept of “topophilia” referring to the affective bond, and he emphasized we have to know places through perception and experience. This affective bond can be either positive or negative to individuals [51]. The positive affective bonds between people and their environment can be formed via the positive perception and experience of places [52] and it can be characterized by the tendency of maintaining a closeness to such places. In terms of green spaces, affective attachment to nature settings has also been referred to as “connectedness to nature”, or a sense of belonging to the natural world [53].

Place identity is “those dimensions of self that define the individual’s personal identity in relation to the physical environment by means of a complex pattern of conscious and unconscious ideas, beliefs, preferences, feelings, values, goals and behavioral tendency and skills relevant to this environment” ([54]; p. 155). Williams *et al.* [48] assumed that a place can become an important part of oneself due to the place satisfying particular needs and goals of oneself, resulting in emotional bonds to the place. In terms of green space, place identity is considered as to what extent residents feel that the urban green space in their neighborhood environment contributes to their identity.

Interactions in places also generate interpersonal relationships [55] through shared meaning and experiences in the places [56]. This social bonding is the most consistent recourse of attachment to places [57]. Urban green spaces can provide vital places where people’s experience can be shared and interpersonal relationships can be formed [58]. Here, we refer to the social bonding dimension of green space as the social relationship maintained through the people-place interactions.

The four-dimensional concept of place attachment has been applied in many different studies, and acceptable levels of reliability and validity have been reported (e.g., [31,45,46]). For example, a survey study among visitors of an Australia’s Great Barrier Reef Marine Park showed that visitors’ evaluation of the park was positively related to their attachment to the park, as measured by the four-dimensional place attachment concept [45]. Another survey among visitors at the Dandenong Ranges National Park, in Australia, revealed that visitors with a high attachment to the park showed a higher intention to engage in pro-environmental behaviors than visitors with a low attachment to the park [46]. To the extent of our knowledge, the four-dimensional concept of place attachment has not yet been applied in the urban residential context to study relationships between attachment to urban green space and health.

### 1.4. The Present Research and Hypotheses

In the present study we examined the impacts of urban green space availability and attachment on residents’ self-reported mental, physical and general health. We selected two neighborhoods that were very similar in the amount of green space as indicated by GIS maps, as well as socio-demographic background and other determinants of well-being, but differed in green space availability. We conducted a survey in which we asked respondents to rate their health, as well as provide other information on their use of nearby green spaces. We hypothesized that: (1) attachment to green space and self-reported health are greater in the neighborhood with higher green space availability; (2) availability of green space is positively related to both green space attachment and self-reported health; and (3) there is a positive path from green space attachment to self-reported health.

## 2. Method

### 2.1. Neighborhood Selection Approach

Groningen is the capital city of a province situated in the north of the Netherlands. It has a population of 195,415 (2013), and administratively the city is divided into 10 Wijk-districts, each district containing four to 10 Buurt-Neighborhoods, yielding 70 neighborhoods in total. Statistics Netherlands indicates a neighborhood is considered to be homogenous in socio-economic structure and function such as residential use, work function, or recreational purposes [59].

In order to test our hypotheses, we selected two neighborhoods that are similar in the physical amount of green spaces, demographic and socio-economic status, and housing conditions, but that differ in green space availability. Following the neighborhood selection procedure used by Van Herzele and De Vries [60], three steps were applied to select the two neighborhoods. 

First, the percentage of urban green spaces in each neighborhood environment was measured using a GIS dataset. For this study, land cover data were used from the Top10NL (the topographic map of the Netherlands, scale 1: 10,000, made in 2013), the most detailed dataset from Dutch National Mapping Agency (Kadaster), which is an objective vector dataset, made from aerial photographs, field surveys and existing files. A 400 m buffer around the domain of the neighborhood was created that presents the land cover of neighborhood environment within a reasonable walkable distance of 10–15 min. This way of measuring the neighborhood green space has been recommended by Saelens *et al.* [61] and Pikora *et al.* [62], and has previously been applied by Leslie *et al.* [63]. We measured the neighborhood green spaces including all public natural areas such as parks, lawns, urban forests and other vegetated land cover. Based on these results, the Groningen neighborhoods were divided into groups with similar percentages of green spaces and total neighborhood environmental sizes.

Second, in order to identify neighborhoods with similar population characteristics and housing conditions we adopted the neighborhood typology procedure elaborated by Vanneste [64] that was applied in Vanneste *et al.* [65] and Van Herzele and De Vries [60]. Thus we identified neighborhoods with similar demographics information (population density, age structure, ethnicity and household structure), socio-economic status (income and car ownership) and housing conditions (percentage of rented accommodation, age of dwellings, per house value and building density within neighborhood buffer area). The census data were derived from the 2011 Statistics Netherlands dataset “*Kerncijfers wijken en buurten*” [59]. With this procedure, only three pairs of neighborhoods met our criteria. 

From these three sets, we evaluated which pair(s) of neighborhoods differed in neighborhood green space availability in terms of accessibility and usability. By means of GIS and field observation we compared accessibility and usability within the neighborhood environment. As suggested in the research of Van Herzele and Wiedemann [66], we took into account the accessibility and usability of the green spaces and the main effects of barriers (e.g., reserved land with bad maintenance, green spaces beside highway with no access). The neighborhoods of De Hoogte and Corpus Den Hoorn-Noord (or “Corpus-Noord”, as we will refer to it in this paper) showed the strongest contrast in neighborhood green space availability.

### 2.2. General Description of the Two Selected Neighborhoods

**Figure 1:**
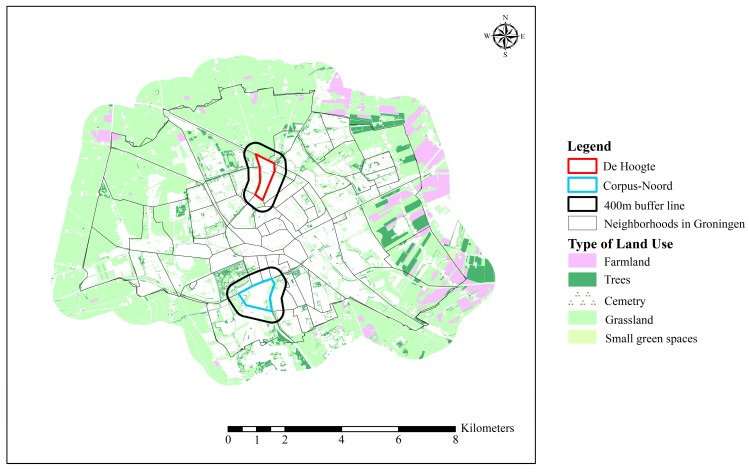
The neighborhoods of De Hoogte (north); and Corpus-Noord (south).

De Hoogte is located in the northern part of Groningen, which was originally built in the 1920s, while Corpus-Noord is in the southern part, built in the 1950s (Figure 1). The types of houses in both neighborhoods are mixed, including flats of four to five floors on average, detached houses, semi-detached houses, and town houses. Flats and houses are usually separated by a green space. Most detached houses and semidetached houses are around two or three story high, usually with a front or back yard garden.

Although our selection procedure identified the two neighborhoods as a relatively similar set within the entire number of neighborhoods in Groningen, there were some notable differences in socio-economic characteristics (Table 1). Among other things, De Hoogte was somewhat more densely populated than Corpus-Noord, and De Hoogte also contained more low income households, and more rental houses. These differences suggest that De Hoogte was of slightly lower socio-economic status than Corpus-Noord, which is indicative of a lower health status of the population. However, De Hoogte also had a somewhat higher percentage of men, and more residents in younger age categories, which could positively influence the health status of the population (since women and the elderly are known to be more prone to bad health). Thus, the socio-demographic differences between the two neighborhoods do not raise concern that there may be large *a priori* differences in health status between the two neighborhoods.

**Table 1:** Characteristics of the two neighborhoods calculated from GIS and census data [59].

Characteristic	De Hoogte	Corpus-Noord
Size of neighborhood with buffer area (in Ha)	265.8	293.3
% of urban green spaces	28.9%	24.7%
population density (persons/Ha)	52.9	47.9
Gender (% female)	46.6%	54.2%
≤24 years	34%	22%
25–44 years	41%	30%
44–64 years	19%	20%
≥65 years	7%	28%
Income (% low)	62%	49%
House ownership (% rented)	93%	69%
Car ownership per household	0.3	0.5
Average distance to supermarket (in km)	0.5	0.4
Average distance to restaurant (in km)	0.4	0.9
Number of secondary schools within 5 km of the home	10	9

### 2.3. Green Space Characteristics

Percentage of green spaces in the neighborhood and its surrounding environment, calculated from GIS, is somewhat lower in Corpus-Noord (24.7%) than in De Hoogte (28.9%). However, the most distinguishing difference, as intended, is that the Corpus-Noord has more accessible and usable green spaces (75%) than De Hoogte (46%). The availability of green spaces was determined through GIS analyses and field observations, carried out by one of the researchers. Green spaces mapped in the GIS were visited and their accessibility and usability were marked and determined (Table 2). If a green space was not fully publicly accessible or lacked using possibilities, it was marked as lacking in availability. For example, a football court is not accessible for residents and a cemetery is aimed at a specific group of users and only has limited opening hours. Leftover green spaces alongside highways or vacant land with weeds were also identified as green spaces with a low use potential.

**Table 2:** Overview of inaccessible and less usable green spaces in the two neighborhoods.

Type	Corpus-Noord		De Hoogte	
	Number	% of Total Green Spaces	Number	% of Total Green Spaces
Less usable green: cemetery	0	0	2	5%
Inaccessible green: sport court	2	20%	0	0
Leftover or undeveloped green (alongside high way)		5%		49%
Total	2	25%	2	54%

**Figure 2:**
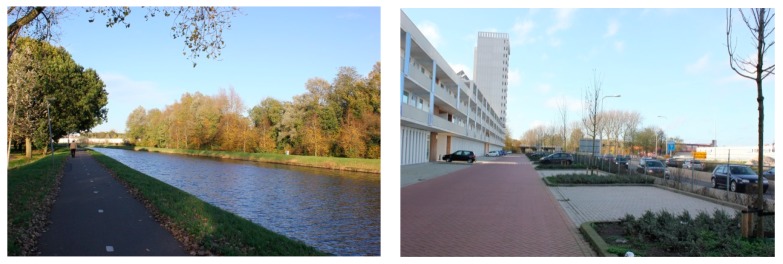
Impressions of green spaces in the two neighborhoods: Green space next to the highway in Corpus-Noord (**left**); and the highway next to the residential area in De Hoogte (**right**).

Both neighborhoods are adjacent to big city parks with Noorderplantsoen park adjoining De Hoogte and Stadspark park in Corpus-Noord, both of which parks are accessible and usable for the residents. With regard to the other surrounding greenspace in the 400 m buffers around the neighborhoods, there are more obstacles in De Hoogte than in Corpus-Noord to reach these spaces. In De Hoogte, a highway crosses the northern part; a railway goes along the west side; and a canal is on the northern side of the neighborhood. Corpus-Noord also contains three main obstacles. A canal together with a highway are adjacent to the east side and another highway is on the northern side of the neighborhood. When looked at in more detail, the canal and the highway in Corpus-Noord maintain a suitable distance from the residential areas. A paved cycling road alongside the canal allows people to access the green spaces and it also provides a good recreational experience for visitors (Figure 2). By contrast, both the highway and railway in De Hoogte are very close to the houses and there are no facilities for them to be traversed, which makes it very difficult for residents to visit the green spaces on the other side of the highway and railway even though physically the green space is nearby.

With respect to green spaces within the two neighborhoods and their surrounding environment, we noted that there were two cemeteries in De Hoogte, which are not open the whole day long, while there was an inaccessible football court in Corpus-Noord. However, as shown in Table 2, by far the highest percentage of inaccessible green was formed by leftover green alongside highways in De Hoogte.

### 2.4. Survey Design

Data were collected by means of paper-mailed questionnaires that were randomly distributed in the two neighborhoods in June 2014. The principal investigator personally visited the neighborhoods and put a questionnaire in the mailbox of every one out of two households. In total, around 2750 questionnaires were distributed this way. Residents received a mail-back envelope and a letter with a questionnaire and a short introduction of the study. The introduction indicated that their responses would be treated anonymously and contained a link to the website address of the online survey. The questionnaire was in Dutch as the majority of households are Dutch, but an English version was available online. A pilot test was done to ensure readability, question clarity and acceptability (*cf.* [60]).

The questionnaire was divided into four parts. The first part consisted of questions on socio-demographic characteristics, including years of education, household net income per month by seven levels (<1000, 1000–2000, 2000–3000, 3000–4000, 4000–5000, 5000–6000, >6000 euro), age, gender, living period in the neighborhood, and frequency of visiting the neighborhood green spaces measured on a five-point scale (seldom or never, monthly, weekly, several times a week and daily).

In the second part of the questionnaire, green space attachment was measured by place dependence (four items), affective attachment (three items), place identity (three items) and social bonding (four items). The questions of place attachment were derived from Kyle, *et al.* [31] and Wynveen *et al.* [45] with a little modification, and were measured on a five-point scale, ranging from “strongly disagree” to “strongly agree”.

The third part consisted of questions on self-reported health and satisfaction with one’s life and neighborhood. Self-reported health was measured by three indicators: mental health, physical health and general health. Mental health was measured by the Mental Health Inventory (MHI-5) from the Short-Form 36 [67], which included five questions on a 6-point scale. Scores on this measure were derived by calculating the average of the responses to the six questions. Physical health was measured with a single question from the SCL-90R [68], saying “In the last month, to what extent have you been bothered by the following 12 physical symptoms: headache, dizziness, chest or heart pain…” Responses were given on a 5-point scale ranging from “not at all” to “very much”. Self-reported general health was estimated by the question “In general, how would you rate your health on a 5-point scale ranging from poor to excellent”. The questions on life- and neighborhood satisfaction are beyond the scope of the present discussion and will not be elaborated on here.

The fourth part of the questionnaire consisted of questions on residents’ perceptions of neighborhood green space and their participation in activities in nearby green space. Most of these questions are not relevant for the present discussion and will be reported elsewhere. Two questions on perceived accessibility of green spaces (“green spaces in my neighborhood are easily accessed, there many access points, enough walking paths, roads around are not busy”) and perceived presence of amenities (“green spaces in my neighborhood provide amenities for sitting, picnic tables, litter bins, sign and lighting at night”) were used as a check on the validity of our GIS analyses of differences in green space availability. Responses to these questions were measured on a 5-point scale ranging from “strongly disagree” to “strongly agree”.

### 2.5. Data Analysis

Confirmatory factor analyses (CFA) were used to assess whether the second-order factor model of the green space attachment with four sub-dimension fits with the data. This was done with the software LISREL 8.80. Differences in self-reported health and green space attachment between the two neighborhoods were tested with one-way MANOVA analysis. If there were significant differences between the neighborhoods in green space attachment or health indicators, a Structural Equation Model (SEM) approach was used to examine the pathways between green space availability, green space attachment and the health variables in the whole sample. Path relationships and coefficients were measured by the maximum likelihood methods in LISREL.

## 3. Results

### 3.1. Descriptive Statistics

In total, 276 questionnaires were returned, of which nine were from the online survey. However, 28 questionnaires were returned empty and another 25 contained too many (>5 per respondent) missing values, leaving 223 questionnaires with (almost) complete data available for analysis. The net responses were 90 in De Hoogte and 133 in Corpus-Noord. The overall response rate is 8.1%, with 7.2% for De Hoogte and 8.7% for Corpus-Noord. Occasional missing values (<1%) were imputed using the Expectation-maximization method in SPSS. 

The socio-demographic characteristics of the two neighborhoods as reported by the respondents are presented in Table 3. The results largely confirm the data from the census as presented in Table 1. There were slightly more households with a low income (and less households with a high income) in De Hoogte than in Corpus-Noord, and the average age of respondents in De Hoogte was lower than in Corpus-Noord. The lower age of respondents may explain the differences in living period between the two samples. The percentage of female respondents in Corpus-Noord was very similar to the census data (55.6% sample *vs.* 54.2% census), but female participants in De Hoogte were apparently more likely to respond to the survey (61.1% sample *vs.* 46.6 census).

**Table 3:** Self-reported socio-demographic statistics of the participants from the two neighborhoods.

Characteristics	De Hoogte	Corpus-Noord	Total
**Socio-demographic characteristics**			
**Respondents**	90	133	223
**Female (%)**	61.1	55.6	42.2
**Average age (years)**	39	49.6	45.4
**Years of education (years)**	16	14.2	14.9
**Household net income (%)**			
low (<1000 euro)	27.8%	17.3%	21.5%
middle (1000–4000 euro)	68.9%	70.7%	70.0%
high (>4000 euro)	3.3%	12.0%	8.5%
**Years of residence in the neighborhood**	8.3%	13.1%	11.2%
**Frequency of visiting green spaces (%)**			
Daily to weekly	63.3%	72.2%	68.6%
Monthly to never	36.7%	27.8%	31.4%

### 3.2. Manipulation Check: Perceived Availability of Green Space

A one-way MANOVA showed that the two neighborhoods differ significantly in perceived accessibility of the green spaces, *F*(1, 221) = 6.33, *p* < 0.05, η*_p_*^2^ = 0.03, and perceived presence of amenities, *F*(1, 221) = 20.32, *p* < 0.001, η*_p_*^2^ = 0.08. Corpus Noord was rated significantly more accessible (*M* = 3.9, *SD* = 0.62) than De Hoogte (*M* = 3.66, *SD* = 0.81). Corpus Noord was also perceived to have more amenities (*M* = 3.37, *SD* = 1.02) than De Hoogte (*M* = 2.74, *SD* = 1.01). These findings are consistent with our GIS analyses of the differences in green space availability between the two neighborhoods.

### 3.3. Green Space Attachment

A second order Confirmatory Factor Analysis (CFA) with maximum likelihood method was applied in LISREL to determine whether the four-dimensional structure of the green space attachment scale was confirmed by the data. As shown in Table 4, all factor loadings, both the first-order and second-order factor loadings, ranged from 0.56 to 0.92, and thus were higher than the acceptable factor loading of 0.5 [69], and were also statistically significant at *p* < 0.001. Consequently, the four-dimensional structure can be confirmed. The composite reliability values of all the five latent variables were higher than the recommended 0.6 [70], which suggests that the five indicators provide reliable measurement of the construct of green space attachment. Indices of the measurement model all met the above goodness-of-fit standards (χ^2^(73) = 154.41, *p* < 0.01, RMSEA = 0.071, SRMR = 0.051, CFI = 0.98, PGFI = 0.63, PNFI = 0.77). Therefore, we can conclude the second-order attachment model had a good model fit.

**Table 4:** Measurement model of second-order factor green space attachment.

Latent Variable Indicators	Factor Loading	*t*-Value	Composite Reliability
Green space attachment			0.83
Place dependence	0.56	7.49	0.84
PD1 I enjoy visiting green spaces in my own neighborhood more than visiting any other green spaces	0.85	-	
PD2 I get more satisfaction out of visiting green space in my own neighborhood than I get from visiting green spaces elsewhere	0.84	13.63	
PD3 I prefer the green space in my own neighborhood over other green spaces for the recreational activities that I enjoy most	0.73	11.62	
PD4 I wouldn’t substitute any other green spaces for the green spaces in my own neighborhood	0.60	9.15	
Affective attachment	0.94	12.39	0.92
AA1 The green spaces in my neighborhood mean a lot to me	0.82	-	
AA2 I am very attached to the green spaces in my living environment	0.91	16.96	
AA3 I feel a strong sense of belonging to green spaces in my living environment	0.92	17.27	
Place identity	0.83	11.89	0.88
PI1 I feel that green spaces in my living environment are part of me	0.90	-	
PI2 I identify strongly with the green space in my living environment	0.89	18.00	
PI3 Visiting green spaces in my living environment says a lot about who I am	0.73	13.20	
Social Bonding	0.60	7.64	0.86
SB1 The time spent in the green spaces in my neighborhood allows me to bond with my family and friends	0.77	-	
SB2 I have a lot of fond memories of past experiences with family in green spaces in my living environment	0.79	11.67	
SB3 Visiting green space in the neighborhood allows me to spend time with my friends and family	0.77	11.31	
SB4 I associate special people in my life with green space in my living environment	0.78	11.52	

### 3.4. Green Space Attachment and Health by Neighborhoods

A one-way MANOVA on the weighted averages of the items loading on the green space attachment dimensions showed that respondents from Corpus-Noord report a significantly higher level of attachment to their neighborhood green spaces than respondents from De Hoogte on all dimensions of attachment (Table 5).

**Table 5:** Mean scores on dimensions of green space attachment and health measures in the two neighborhoods (range 1–5, standard deviations between brackets).

Variable	De Hoogte (Low Green Space Accessibility/Usability) (*n* = 90)	Corpus-Noord (High Green Space Accessibility/Usability) (*n* = 133)	*F*	*p*	η*_p_*^2^
**Place Dependence**	2.34 (0.74)	2.66 (0.74)	9.82	0.002	0.043
**Affective attachment**	2.62 (0.88)	2.95 (0.77)	9.00	0.003	0.039
**Place Identity**	2.81 (1.08)	3.23 (0.94)	9.40	0.002	0.041
**Social Bonding**	1.91 (0.78)	2.30 (0.70)	14.87	0.000	0.063
**Mental Health**	4.65 (0.75)	4.89 (0.57)	7.79	0.006	0.034
**Physical Health**	3.96 (1.02)	4.02 (0.91)	0.24	0.629	0.001
**General Health**	3.26 (1.0)	3.18 (0.90)	0.34	0.558	0.002

Consistent with our expectations, the mean value of mental health in De Hoogte was significantly lower than that of respondents in Corpus-Noord, mean difference = −0.24. There were, however, no significant differences between the neighborhoods in the mean values of physical and general health. Given the significant differences in mental health between the two neighborhoods, it is possible that these differences are related to green space availability and green space attachment. In the next step, a structural equation model was set to measure the relationship between green availability, green space attachment and mental health.

### 3.5. Structural Equation Model 

A SEM was specified to estimate the relationship between green space availability, green space attachment and mental health while taking into account the influences of socio-demographic status, frequency of visiting neighborhood green spaces and length of residence in the neighborhood. In the literature, length of residence and visiting frequency have been considered to be predictors of place attachment, and age, education and income show erratic patterns of associations with place attachment [38]. The socio-demographic status may moderate the impacts of green space availability on mental health. Green space availability, as indicated by the neighborhood, was included in the model as a dummy variable. We used maximum likelihood method to estimate each path relationship.

**Figure 3:**
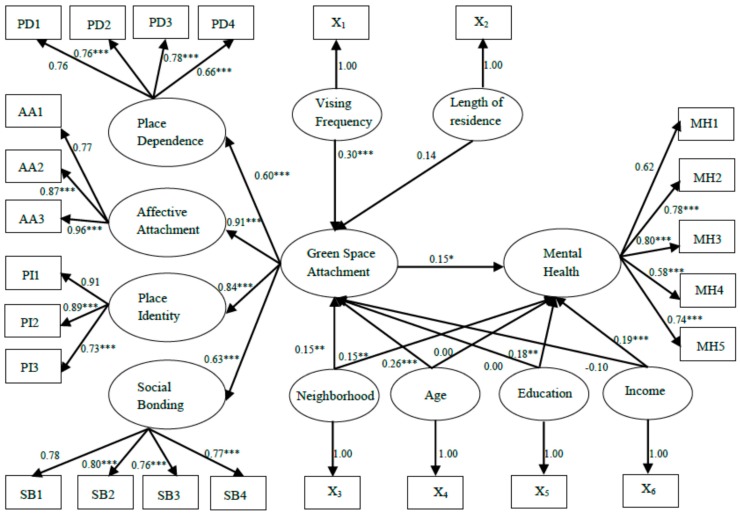
Standardized results of the structural equation modelling, * *p* < 0.10, ** *p* < 0.05, *** *p* < 0.01. Note: PD1-PD4, AA1-AA3, PI1-PI3, and SB1-SB4 are indicators of the four latent variables of green space attachment shown in Table 4, and MH1-MH5 refer to indicators of mental health. X_1_–X_6_ are a set of exogenous variables which correspond to visiting frequency, length of residence, neighborhood, age, education and income.

The results of the SEM are presented in Figure 3. The model fit-statistics suggest a good fit: (χ^2^(245) = 329.45, *p* < 0.001, RMSEA = 0.039, SRMR = 0.058, CFI = 0.98, PGFI = 0.67, PNFI = 0.77). In line with the findings of the CFA, the measurement model suggests a good fit between the data and the latent constructs of green space attachment and mental health. The composite reliability of the six endogenous latent variables was higher than the required 0.6. These results confirm that the measurement model is a reliable construct and has a good discriminate validity.

The structural model reports the relationships among the latent variables and the relationships between latent variables and exogenous variables. Consistent with the previously found differences between the neighborhoods, neighborhood (as an indicator of availability of green space) has a positive and significant influence on green space attachment, β = 0.15, *t*(245) = 2.16, *p* < 0.05, as well as on mental health, β = 0.15, *t*(245) = 2.10, *p* < 0.05. Furthermore consistent with our expectations, there is a marginally significant relationship between green space attachment and mental health, β = 0.15, *t*(245) = 1.89, *p* = 0.06. Age and green space visiting frequency are significant and positive predictors of emotional attachment to green space with coefficients of 0.26 and 0.30, respectively. This indicates that older people tend to be more attached to neighborhood green spaces than younger people, and that increasing the visiting frequency helps to foster a sense of green space attachment. In contrast to our expectation, although the coefficient is positive, β = 0.14, the length of residence does not significantly impact green space attachment, *t*(245) = 1.60, *p* > 0.11. Green space attachment is also not significantly related to years of education, *p* > 0.99, and income, *p* > 0.12. Years of education, β = 0.18, *t*(245) = 2.58, *p* < 0.05, and income, β = 0.19, *t*(245) = 2.72, *p* < 0.01, are positively related to mental health. However, there is no significant association between age and mental health, β = 0, *t*(245) = 0.03, *p* > 0.97).

## 4. Discussion

This study provides empirical support for the idea that besides the quantity of urban green space, the availability of, and attachment to, green spaces may contribute to its health effects. We performed a comparative study in two urban neighborhoods in a Dutch city that are quite similar in the amount of green spaces, socio-demographic status and other environmental characteristics, but differ in the availability of accessible and usable green space, as indicated by GIS data, field observations and residents’ perceptions. By means of a survey we collected data on residents’ attachment to green space and their self-reported general, physical and mental health. Structural equation modelling was used to further examine differences in green space attachment and mental health between the two neighborhoods.

### 4.1. Green Space Availability and Green Space Attachment

Our findings show that residents of the neighborhood with high availability of accessible and usable green spaces report stronger attachment to green space in their neighborhood than residents of the neighborhood with low availability of such green spaces on each of four dimensions of green space attachment: place dependence, affective attachment, place identity and social bonding. These relationships are confirmed in a Structural Equation Model, controlling for the influence of socio-demographic variables. A relationship between availability of, and attachment to, green space is consistent with previous research showing that place attachment may be facilitated by physical characteristics like proximity and accessibility [40]. However, to our knowledge, this is the first study in which attachment to green spaces in particular has been linked to physical characteristics of those green spaces.

Theoretically, a link between green space availability and attachment may be explained by the fact that residents in neighborhoods with accessible and usable green space have more and/or longer or otherwise more intensive interactions with the green spaces, thus providing them with more opportunities to become connected to the green space [58]. This explanation was not supported by our data, as residents of the two neighborhoods reported similar visiting frequency to the green spaces in their neighborhood. It should be noted, however, that visiting frequency was measured with only one single question, which may not have been sensitive to capture subtle differences in contact with green space between the two neighborhoods in terms of staying time and the kind of activities performed.

### 4.2. Health Differences

Residents in the neighborhood with a high availability of accessible and usable green spaces reported significantly better mental health than residents in the neighborhood with a low availability of such spaces. This finding is consistent with the idea that direct experience with nature is a powerful way of obtaining relief from stress and other mental problems [71]. Residents in the neighborhood with a high availability of accessible and usable green spaces presumably have more such opportunities than residents in the neighborhood where a large percentage of the green space is unusable or inaccessible.

Prevailing theories state that contact with green space improves mental health via the recovery of attention fatigue and stress [72,73]. Alternatively, it has been suggested that mental health effects of green space may derive from residents’ emotional attachment, or connectedness, to green spaces [74]. In the present study, mental health was indeed weakly, but positively, related to green space attachment. This finding resonates with a growing literature highlighting the importance of a sense of connectedness to the natural world for people’s wellbeing and health [75]. In addition, the positive association between green space attachment and mental health corroborates the theoretical assumption that place attachment leads to improved wellbeing (see [32,38]). However, this attachment may be also formed by repeated positive, healthy and restorative, experiences with green spaces in people’s everyday lives. Therefore, it might be possible that better mental health fosters a sense of green space attachment, instead of green space attachment fostering better mental health. Such reverse causality cannot be excluded based on the present, correlational, data.

Self-reported physical and general health did not significantly differ between the two neighborhoods. These findings are largely consistent with previous research, which have also found that green space is more important for mental health than for general and physical health [76,77]. In neighborhoods with a low green space availability the lack of accessible and usable green spaces might be complemented by other neighborhood characteristics such as walkable streets and playgrounds to support walking, jogging and other physical activities, which are known to contribute to general and physical health.

### 4.3. Other Predictors of Green Space Attachment and Mental Health

In the present study we developed a new measure of green space attachment based on previous four-dimensional conceptualizations of place attachment [31,45,46]. Besides associations with green space availability and mental health, ratings of green space attachment were also significantly and meaningfully related to several socio-demographic characteristics. Importantly, we found that frequency of visits to neighborhood green space is significantly positive related to green space attachment. A case study in the Netherlands also found that the more often people visit an urban park, the more connected and attached they are to it [58]. Our data also show that older residents are more likely to attach to green space than younger people. This may be because older people are less mobile than young people, and may have more time to visit the green spaces in their neighborhood. Education and income were not associated with green space attachment but were positively related to mental health. These findings suggest that people with a higher socio-economic status may have more personal resources to maintain and regulate their mental health, and are therefore less dependent on neighborhood resources [78]. Length of residence, although positively related to green space attachment, was not a significant predictor of place attachment. Previously, Scannell and Gifford [44] also did not find significant relationships between length of residence and natural place attachment (a concept similar to green space attachment).

### 4.4. Limitations and Suggestions for Future Research

The main strength of the present study is that it combines insights from geography and psychology to understand health effects of neighborhood green space. Nevertheless, the research is not without limitations. A first limitation is that, despite our careful precautions to select neighborhoods that were similar in all respects but green space availability, we noticed some differences between the two neighborhoods in the sample data as well as compared with the census data on social-demographic characteristics (e.g., mean age of the two neighborhoods). Although the main socio-demographic variables have been taken into account in the SEM, it cannot be ruled out that part of the neighborhood differences in green space attachment and mental health were caused by differences in the social-demographic composition of the neighborhoods. A second limitation is that we only used self-report measures of health, we did not use medical records or other more objective measures of health. Although green space availability was derived from a different data source, health ratings, green space attachment and other variables are provided by the same source. Using same-source data may generate spurious association between variables [16]. The restriction to two neighborhoods in one Dutch city may limit the generalizability of the results to other neighborhoods and cities. We did not obtain detailed measurements or observations of resident’s interactions with the green spaces in their neighborhood, and thus were unable to fully address the potential influences of this variable. Finally, because all our data are correlational, causal influences suggested by the path model must remain tentative.

For future research, we suggest qualitative research may help to explore in-depth how people form intimate relationships with green spaces in their neighborhoods. Further efforts are also warranted to obtain objective measurements of health and interactions with green space in relation to green space attachment. Given the promising results of the present study, it would also be worthwhile to study relationships between green space availability, attachment and health in more large-scale epidemiological studies with large numbers of different types of neighborhoods. By including different types of neighborhoods (e.g., low income, middle income, and high income), it is possible to explore moderating influences of social-demographic composition of neighborhoods on the relationships between green space availability, attachment and mental health. This is important for understanding the critical thresholds of neighborhood contexts in the formulation of green space attachment and health.

## 5. Conclusions and Policy Implications

In the present study, we found that residents of two urban neighborhoods that were similar in green space quantity (and other health-indicating characteristics) but differed in the availability of those green spaces, showed significant discrepancy in their mental health condition and green space attachment. Secondly, green space attachment seems to be relevant in fostering a healthy mental status. As a consequence, we recommend that the provision of urban green spaces should not only consider the amount of green spaces but also their accessibility and usability. Setting quantified standards in providing urban green spaces may cause imbalances in access to green spaces due to the discrepancies of green space accessibility and quality. Given that health impacts of green space may differ depending on their availability, green space policy and planning need to target on insuring that everyone possesses the equal opportunity to enjoy urban green spaces.

In short, as implications for planning practice, we suggest that urban green space policy and planning should combine both quantified goals and qualitative standards in providing neighborhood green spaces. We hope that the findings of our study will encourage decision makers and planners to move out of the familiar realm of setting quantitative standard to the realm of qualitative analysis. We also hope that our findings will stimulate researchers to conduct more research on understanding how green space attachment formed by daily experience may impacts on the health and wellbeing of residents. We should not make green spaces, but green places.
